# Levels of positive aspects of caregiving and associated factors among family caregivers of older adults: a systematic review and meta-analysis

**DOI:** 10.3389/fpubh.2026.1844907

**Published:** 2026-06-12

**Authors:** Ling-Na Kong, Dun-Xiu Liu, Jun Yang, Li Yang, Ping Hu

**Affiliations:** 1 Department of Nursing, The First Affiliated Hospital of Chongqing Medical University, Chongqing, China; 2 School of Nursing, Chongqing Medical University, Chongqing, China; 3 International Medical Center, The First Affiliated Hospital of Chongqing Medical University, Chongqing, China; 4 Department of General Practice, The First Affiliated Hospital of Chongqing Medical University, Chongqing, China; 5 School of Nursing, Qingdao University, Qingdao, China; 6 Department of Neurology, The First Affiliated Hospital of Chongqing Medical University, Chongqing, China

**Keywords:** associated factors, family caregivers, informal care, meta-analysis, older adults, positive aspects of caregiving, systematic review

## Abstract

**Objectives:**

To systematically assess the levels of positive aspects of caregiving, measured by validated scales, and examine associated factors among family caregivers of older adults.

**Design:**

A systematic review and meta-analysis.

**Methods:**

PubMed, CINAHL, Embase, and Web of Science were searched from inception to 30 August 2025. Two reviewers conducted study selection, quality appraisal, data extraction, and data analysis. The mean scores of positive aspects of caregiving were synthesized using the random-effects model. Associated factors were summarized using the stress process model as the theoretical framework.

**Results:**

A total of 34 studies were included, involving 8,021 family caregivers from 12 countries. The pooled mean score for positive aspects of caregiving was 3.50 (95%CI = 3.32–3.67). Subgroup analyses showed lower positive aspects of caregiving in Asian and younger family caregivers. Associated factors included socio-demographic factors, primary stressors, secondary stressors, and social factors.

**Conclusion:**

Positive aspects of caregiving are at a moderate level among family caregivers of older adults and can be influenced by multifaceted factors. Healthcare professionals should develop support interventions based on modifiable factors to enhance positive aspects of caregiving and improve psychological health for family caregivers.

**Systematic review registration:**

PROSPERO CRD420251154778.

## 1 Introduction

With the increasing aging population, informal care needs and intensity of older adults will continue to grow worldwide (1). Family caregivers are an important force in informal care for older adults and play a critical role in supporting long-term care (2, 3). Family caregivers are unpaid family members who provide primary care and assistance for older adults, such as spouses, adult children, and other relatives (4). In the home environment, caring for older adults with chronic conditions or disabilities is considerably demanding and stressful for family caregivers. It is a high priority to effectively promote the adaptation of family caregivers to the caregiving process (3).

Negative and positive experiences coexist in the adaptation process of caregiving. Family caregivers often experience depressive symptoms and a declined quality of life, which may compromise the caregiving quality (2). Meanwhile, informal caregiving can be beneficial and rewarding. Positive aspects of caregiving (PAC) refer to the satisfaction and rewards derived from the caregiving role (5). PAC encompasses the sense of accomplishment, relationship gains, competence and mastery, personal growth, and role satisfaction (3, 6). PAC has beneficial effects on both caregivers and care recipients. PAC was found to decrease caregivers’ burden (7), depressive symptoms (8), psychological distress (9), and suicidal ideation (10). Moreover, PAC can significantly increase caregivers’ life satisfaction (11), well-being (12), and continuation of caregiving (13). Accordingly, promoting PAC is important to improve both caregivers’ health and the quality of informal care for older adults.

Assessment of PAC and associated factors can help identify vulnerable caregivers and develop targeted interventions. Several self-reported scales are available for measuring PAC for family caregivers, such as the Caregiving Satisfaction Scale (CSS), Positive Aspects of Caregiving Scale (PACS), Gain in Alzheimer Care Instrument (GAIN), and Positive Appraisal Scale (PAS). These scales were developed to assess the positive experience of caregiving, with different dimensions, items, and Likert point scales. For instance, the CSS is one subscale of the Caregiving Appraisal Scale developed by American researchers to assess caregivers’ positive and negative appraisals of caregiving (14). The CSS has six items rated on a 5-point Likert scale. The PACS, a 5-point Likert scale, was adapted from the CSS (5). It comprises two dimensions: self-affirmation and outlook on life. The PACS has several versions, such as 11-item, 9-item, 7-item, and 6-item versions. Due to satisfactory reliability and validity, these scales have been used in research on family caregivers. Their measurements can inform levels of PAC and interventions to improve PAC.

With the development of positive psychology, PAC have received greater attention. A growing body of studies have focused on evaluating PAC using validated scales among family caregivers of older adults and found different levels of PAC. In addition, these studies discovered a variety of factors related to PAC, such as demographic, psychological, and social (11, 15, 16). As an amount of evidence continues to emerge on this topic, a systematic review is needed to clarify the current understanding of PAC and its associated factors. Nevertheless, a limited systematic review focuses on synthesizing quantitative studies on assessing levels of PAC and associated factors in this population. According to the Stress Process Model, the caregiving stress process consists of four domains: background and context, primary and secondary stressors, stress mediators, and stress outcomes (17). Therefore, this systematic review aimed to (1) estimate the levels of PAC measured by validated scales among family caregivers of older adults and (2) summarize factors associated with PAC, using the Stress Process Model as the theoretical framework. The findings will provide evidence for supportive interventions for family caregivers of older adults.

## 2 Methods

### 2.1 Design

This systematic review was reported following the Preferred Reporting Items for Systematic Reviews and Meta-Analyses (PRISMA) guidelines (18). The review was registered on the PROSPERO (CRD420251154778).

### 2.2 Search strategy

PubMed, CINAHL, Embase, and Web of Science were searched from inception to 30 August 2025, with language restriction to English. Search terms were MeSH terms and free terms related to PAC and caregivers, such as positive aspect* of caregiving, caregiving gain*, benefit finding, caregiving appraisal*, caregiving satisfaction, caregivers, caregiver*, and carer*. The search strategy is presented in Supplementary Table 1. Moreover, references of included studies were manually screened to identify additional studies.

### 2.3 Inclusion and exclusion criteria

The inclusion criteria were (1) population: unpaid family caregivers of older adults (≥ 60 years), such as adult children, spouses, or other relatives; (2) outcome: PAC measured by validated scales; (3) setting: community or home; and (4) study design: cross-sectional or longitudinal study. The exclusion criteria were as follows: (1) qualitative studies, intervention studies, reviews, conference abstracts, letters, or editorials; (2) studies involving non-family caregivers, such as friends or neighbors; (3) studies involving care recipients aged less than 60 years; (4) studies involving institutionalized care recipients; (5) studies without relevant data to calculate mean scores of PAC; and (6) studies with duplicate data.

### 2.4 Study selection and data extraction

One reviewer conducted study selection and data extraction, and then another reviewer checked the accuracy. Disagreements were settled by discussion or consultation with a third reviewer. For study selection, records from the database search were imported into EndNote X9, and duplicates were removed. After removing irrelevant studies by screening titles and abstracts, the full texts of the remaining studies were assessed to identify eligible studies. Extracted data were as follows: (1) study characteristics: author, publication year, country, study design, recruitment time, and sample size; (2) caregivers’ characteristics: mean age, gender, and type of caregivers; (3) care recipients’ characteristics: age and health conditions; and (4) outcomes: outcome measures, mean scores of PAC, and associated factors.

### 2.5 Quality appraisal

Two reviewers independently conducted the quality appraisal. Discrepancies were resolved by discussion with a third reviewer. The quality of included studies was assessed using the Agency for Healthcare Research and Quality (AHRQ) for cross-sectional studies (19). The AHRQ contains 11 items. Each item is scored 1 for an answer of “yes” and 0 for an answer of “no” or “unclear.” The total score of 0–3, 4–7, and 8–11 indicates low, moderate, and high quality, respectively.

The Grading of Recommendations Assessment, Development and Evaluation (GRADE) system (20) was used to assess the quality of evidence. The quality of evidence is classified as high, moderate, low, or very low. Observational studies are initially rated as low quality. The quality rating can be downgraded or upgraded according to specific criteria.

### 2.6 Statistical analysis

Meta-analyses were conducted using Stata 12.0. Mean scores of PAC were pooled by weighted effect size with 95% confidence intervals (CI). The random-effects model was chosen for meta-analysis when significant heterogeneity was present (*p* value for Cochran’s Q < 0.10 or I^2^ statistic > 50%); otherwise, the fixed-effects model was utilized. Subgroup analyses for mean scores of PAC was undertaken to investigate potential sources of heterogeneity according to the following variables: geographical region (Asia vs. Europe vs. North America vs. Australia), participant recruitment time (2010–2019 vs. 2020–2024), mean age of caregivers (< 60 years vs. ≥ 60 years), proportion of female caregivers (< 70% vs. ≥ 70%), proportion of adult-child caregivers (< 50% vs. ≥ 50%), and assessment tools (PACS vs. CSS vs. other scales). Sensitivity analysis was conducted to examine the reliability of the pooled mean scores of PAC. Begg’s test and Egger’s test were used to evaluate publication bias. Due to heterogeneity in assessment tools, data reporting formats, and limited statistical data, factors associated with PAC were summarized narratively based on the Stress Process Model.

## 3 Results

### 3.1 Search results

**Figure 1:**
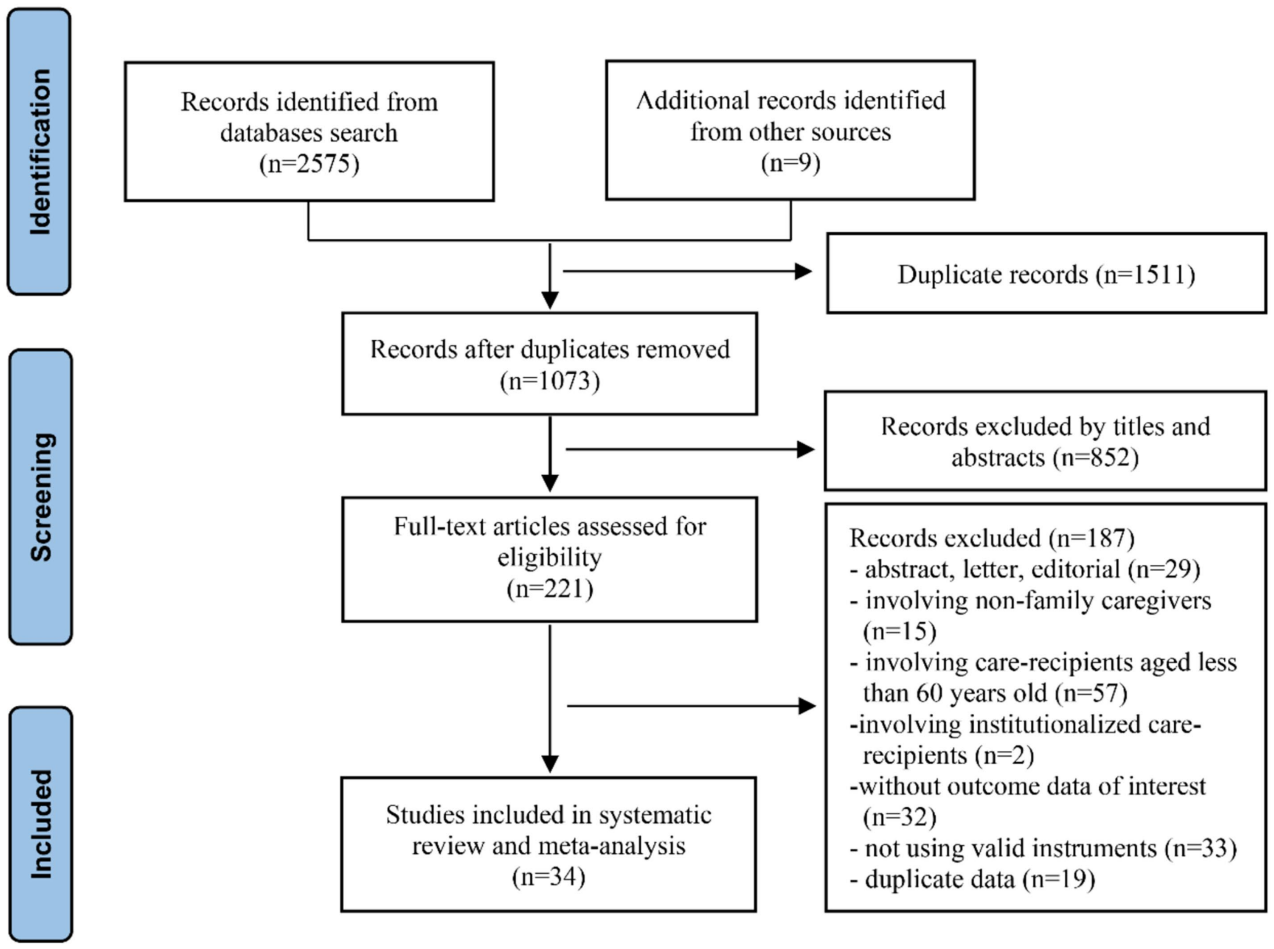
Flow diagram of study selection.

A total of 2,584 records were identified from database search and reference lists. After removing 1,511 duplicates, 1,073 records were screened by titles and abstracts, and 852 records were removed. Of the remaining 221 studies for full-text assessment, 34 studies were included. Figure 1 presents the detailed selection process.

### 3.2 Study characteristics

Characteristics of included studies are shown in Table 1. They were published between 1999 (21) and 2025 (22), and 19 (55.9%) studies were published between 2020 and 2025. Of the included studies, 23 (67.6%) were conducted in Asia, 7 (20.6%) in Europe, 3 (8.8%) in North Ameira, and 1 (2.9%) in Australia. All studies used a cross-sectional design. These studies involved 8,021 family caregivers from 12 countries, with sample sizes ranging from 50 (23) to 983 (24). The mean age of family caregivers varied from 46.8 (12) to 78.1 (11) years. The proportion of female family caregivers and adult-child caregivers ranged from 36.9% (25) to 100% (26) and from 40.9% (12) to 100% (27), respectively. All care recipients were aged 60 years or above, with a mean age ranging from 67.28 (24) to 86.7 (16) years. For care recipients’ health conditions, 14 (41.2%) studies focused on dementia (2, 15, 22, 23, 25, 26, 28–35), 6 on dependent older adults (9, 26, 28, 36–38), 5 on frail older adults (11, 27, 33, 39, 40), 4 on disability or functional impairment (7, 15, 21, 35), 2 on older adults requiring care (24, 41), 1 on chronic conditions (16), 1 on heart failure (42), and 1 did not report this information (43). Eleven scales were used to measure PAC in the included studies, among which the PACS were the most commonly used (*n* = 17, 50%). These scales used different item rating criteria, such as 1–5 points, 0–4 points, 1–4 points, and 0–3 points, with higher total scores indicating greater levels of PAC.

**Table 1:** Characteristics of included studies.

Author (year)	Country	Recruitment time	Sample size	Caregivers			Care recipients	Scales (item rating)	Item mean score
				Mean age	% female	% main caregivers			
Alonso et al. (28)	Spain	-	140	61.78	67.9	53.6% adult child	≥ 60 years, dependent older adults	CSS (1–4)	1.79
Chan et al. (39)	Singapore	Jul-Dec 2020	188	62.0	64.9	59.6% adult child	≥ 65 years, frailty and multimorbidity	PACS-7 (1–5)	4.00
Chaudhry et al. (22)	Singapore	2018–2021	169	56.4	69.8	85.8% adult child	Mean age of 82.6 years, dementia	GAIN (0–4)	3.29
Cheng et al. (29)	China	-	99	59.88	71	55% adult child	≥ 60 years, Alzheimer’s Disease	PACS-9 (1–5)	2.77
Gonçalves-Pereira et al. (44)	Portugal	-	116	56.1	67.2	55.2% adult child	Mean age of 77.6 years, dementia	PACS-11 (1–5)	3.61
Huo et al. (10)	China	2019–2022	264, 68	59.1, 60.8	59.6, 68.7	52.2% spouse, 31.3% spouse	Mean age > 70 years, dementia	PACS-9 (1–5)	3.38, 3.66
Iecovich (27)	Israel	-	335	55.79	55.1	100% adult child	Mean age of 84.49 years, frail older adults	CSS (1–5)	4.09
Imasio (41)	Japan	Apr 2010-Feb 2011	192	65.7	76	43.2% spouse	≥ 65 years, requiring care above level 3	PAS (1–4)	2.79
Jiang et al. (11)	China	2016	272	78.1	66.5	100% spouse	Mean age of 81 years, frail older adults	PACS-11 (0–4)	2.28
Kajiwara et al. (13)	Japan	Mar 2010-Nov 2011	343	63.9	79	44.3% adult child	Mean age of 84.7 years, dementia	CGS (0–3)	1.70
Lee and Singh (40)	South Korea	-	137	64.72	78.1	54.8% adult child	≥ 60 years, frail older adults	ACS (1–5)	3.35
Liu et al. (45)	China	-	96	60	72.9	46.9% spouse	≥ 60 years, dementia	PACS-9 (1–5)	3.78
Liu and Sun (15)	China	Oct 2022-Jun 2023	218	57.46	42.66	53.21% spouse	Mean age of 70.67 years, disability	BFS (1–5)	2.54
Liu et al. (31)	China	Mar 2017-Mar 2018	109	65.18	58.7	59.5% spouse	Mean age of 76.02 years, dementia	PACS-9 (1–5)	3.27
López et al. (36)	Spain	-	111	59.7	82	66.7% adult child	Mean age of 82.06 years, dependent older adults	CSS (1–5)	3.73
López-Martínez et al. (9)	Spain	2013–2015, 2015–2016	332	56.27	86.4	74.2% adult child	Mean age of 85.2 years, dependent older adults	CSS (1–5)	4.45
Lou et al. (8)	China	Oct 2020-Aug 2021	328	55.28	81.41	91.62% adult child	Mean age of 85.25 years, dementia	PACS-11 (1–5)	3.28
McAuliffe et al. (32)	Australia	-	134	72.32, 53.36	67.1, 98.2	59.0% spouse	≥ 65 years, dementia	FACQ (1–5)	3.75, 3.82
Narayan et al. (23)	USA	-	50	73.3	74	100% spouse	Mean age of 76.7 years, dementia	PACS-11 (1–5)	3.63
Sabatini et al. (46)	UK	2015–2017	136	53.67	83.1	100% adult child	Mean age of 82.76 years, dementia	PACS-9 (1–5)	3.33
Schulz et al. (37)	Germany	-	270	56.08,55.25	0, 100	100% adult child	≥ 60 years, dependent older adults	PACS-6 (1–5)	2.69, 3.23
Schwarz (21)	USA	-	100	64.7	74	64% spouse	Mean age of 76.5 years, function impairment	CSS (1–5)	4.12
Schwarz and Elman (42)	USA	-	128	64.8	74.2	61.7% spouse	Mean age of 77.3 years, heart failure	CSS (1–5)	4.28
Soskolne et al. (26)	Israel	2003	101,100	68.4, 48.1	100	50% wife, 50% daughter	≥ 65 years, dependent older adults	CSS (1–5)	3.07, 3.34
Sugihara and Sugisawa (24)	Japan	2013–2019	983	67.3	71.1	-	Mean age of 67.28 years, requiring care level 1–5	CPAS (0–4)	2.23
Toljamo et al. (43)	Finland	2002–2004	209	58	74	65% adult child	≥ 65 years	COPE (1–5)	4.06
Tomita et al. (16)	Japan	Nov 2022-Mar 2023	251	66.6	77	-	Mean age of 86.7 years, chronic conditions	PAC (0–3)	2.70
Wang et al. (47)	China	Sep 2022-May 2023	237	62.43	69.1	-	Mean age of 76.24 years, dementia	PACS-9 (1–5)	3.12
Wong et al. (33)	China	-	170	75.65	71.2	100% spouse	Mean age of 79.59 years, frail older adults	PACS-9 (1–5)	3.92
Wu et al. (12)	China	-	132	46.8	68.2	40.9% adult child	Mean age of 73.9 years, dementia	PACS-9 (0–4)	3.28
Yan et al. (38)	China	Jul-Aug 2021	210	73.38	63.59	100% spouse	≥ 60 years, dependent older adults	PACS-9 (1–5)	2.41
Yu et al. (7)	China	Jan-Apr 2023	494	-	61.9	43.7% adult child	≥ 60 years, disability	PACS-9 (1–5)	3.55
Zhang et al. (35)	China	Jul-Aug 2021	601	53.05	63.26	100% adult child	≥ 60 years, physical impairment	PACS-9 (1–5)	3.24
Zhang et al. (25)	Japan	Jun 2024	198	55.48	36.9	74.7% adult child	Mean age of 85.1 years, dementia	PAS (1–4)	2.23

ACS: Appraisal of caregiving scale; BFS: Benefit finding scale; CGS: Caregiving gratification scale; COPE: Carers of older people in Europe; CPAS: Caregivers personal attainment scale; CSS: Caregiving satisfaction scale; FACQ: Family appraisal of caregiving questionnaire; GAIN: Gain in Alzheimer care instrument; PAC: Positive aspects of caregiving; PACS: Positive aspects of caregiving scale; PAS: Positive appraisal scale.

### 3.3 Quality appraisal results

Of 34 studies, 9 studies (7, 8, 13, 16, 24, 35, 39, 41, 43) were appraised as high quality with 8 points, and 25 studies were moderate quality with 5–7 points. Details of quality appraisal for included studies are shown in Supplementary Table 2.

### 3.4 Pooled levels of PAC

**Figure 2:**
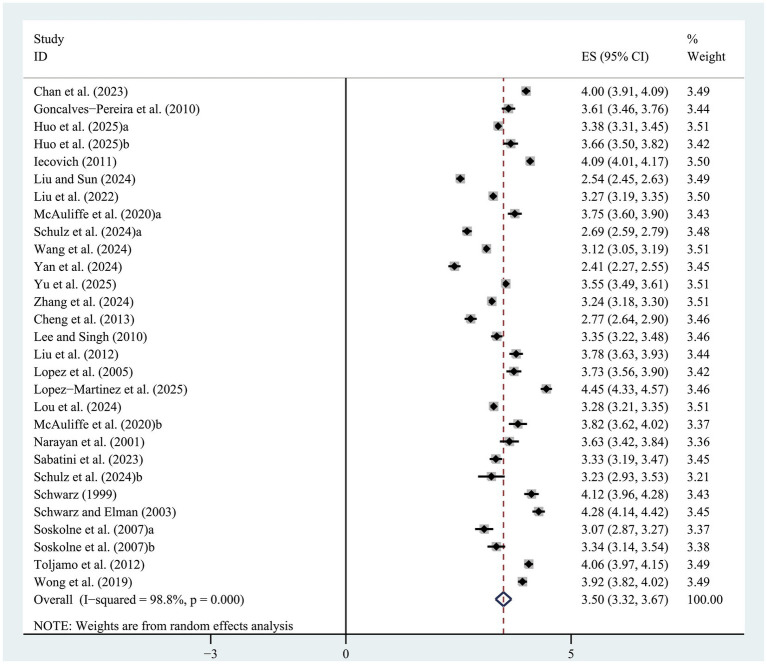
Meta-analysis of mean scores of positive aspects of caregiving measured by scales using item rating of 1–5 points.

Twenty-five studies with 5,341 family caregivers were identified to measure PAC using the CSS (9, 21, 26, 27, 36, 42), PACS (7, 8, 10, 23, 29, 31, 33, 35, 37–39, 44–47), Carers of Older People in Europe (COPE) Index (43), Family Appraisal of Caregiving Questionnaire (FACQ) (32), Benefit Finding Scale (BFS) (15), and Appraisal of Caregiving Scale (ACS) (40) with item rating 1–5 points. Of them, four studies reported two groups of data, respectively (10, 26, 32, 37). High heterogeneity was present between studies (I^2^ = 98.8%, *p* < 0.0001). In a random-effects model, the pooled mean score was 3.50 (95%CI = 3.32–3.67) (Table 2; Figure 2), indicating a moderate level of PAC.

**Table 2:** Meta-analysis of mean scores of positive aspects of caregiving.

Item rating	No. of data	No. of caregivers	Pooled mean scores	Heterogeneity	
			(95%CI)	I^2^ (%)	*p*
Scales with 1–5 points^a^	25	5,341	3.50 (3.32, 3.67)	98.8	< 0.0001
Scales with 0–4 points^b^	4	1,556	2.77 (2.15, 3.39)	99.5	< 0.0001
Scales with 1–4 points^c^	3	530	2.27 (1.74, 2.81)	98.6	< 0.0001
Scales with 0–3 points^d^	2	594	2.20 (1.22, 3.18)	99.6	< 0.0001

a Appraisal of caregiving scale, caregiving satisfaction scale, carers of older people in Europe index, family appraisal of caregiving questionnaire, benefit finding scale, positive aspects of caregiving scale.

b Caregivers personal attainment scale, positive aspects of caregiving scale, gain in Alzheimer care instrument.

c Caregiving satisfaction scale, positive appraisal scale.

d Appraisal of caregiving scale, positive aspects of caregiving.

Four studies with 1,556 family caregivers were found to report PAC measured by the PACS (11, 12), GAIN (22), and Caregivers Personal Attainment Scale (CPAS) (24) with item rating of 0–4 points. A random-effects model was used due to significant heterogeneity (I^2^ = 99.5%, *p* < 0.0001). The pooled mean score was 2.77 (95%CI = 2.15–3.39), suggesting a moderate level of PAC (Table 2; Supplementary Figure 1). Three studies with 530 family caregivers reported PAC using the CSS (28) and PAS (25, 41) (item rating 1–4 points). Using the random-effects model (Heterogeneity: I^2^ = 98.6%, *p* < 0.0001), the pooled mean score was 2.27 (95%CI = 1.74–2.81) (Table 2; Supplementary Figure 2). Additionally, two studies with 594 family caregivers reported PAC using the Caregiving Gratification Scale (CGS) (13) and PAC (16) (item rating 0–3 points). The pooled mean score was 2.20 (95%CI = 1.22–3.18) using the random-effects model (Heterogeneity: I^2^ = 99.6%, *p* < 0.0001) (Table 2; Supplementary Figure 3).

### 3.5 Subgroup analyses

Subgroup analyses were conducted for mean scores of PAC measured by scales using item rating of 1–5 points. As shown in Table 3, family caregivers in Asia (3.34) reported a lower pooled mean score than caregivers in Europe (3.59), Australia (3.78), and North America (4.02). For recruitment time, the pooled mean score was higher in studies that recruited caregivers between 2010 and 2019 (3.59) than in studies between 2020 and 2024 (3.24). In terms of caregiver characteristics, caregivers with a mean age of < 60 years (3.44) had a lower pooled mean score than those with a mean age of ≥ 60 years (3.57). The pooled mean score in studies with more than 70% of female caregivers (3.64) was higher than that in studies with less than 70% of female caregivers (3.33). Besides, the pooled mean score was higher in studies with ≥ 50% adult-child caregivers (3.55) compared to that in studies with < 50% adult–child caregivers (3.47). Regarding scales, the pooled mean score was 3.35 in studies using the PACS, 3.88 in studies using the CSS, and 3.50 in studies using other scales.

**Table 3:** Subgroup analyses for mean scores of positive aspects of caregiving measured by scales using item rating of 1–5 points.

Subgroups	No. of data	No. of caregivers	Pooled mean scores	Heterogeneity	
			(95%CI)	I^2^ (%)	*p*
Geographical region					
Asia	17	3,755	3.34 (3.14, 3.54)	98.8	< 0.0001
Europe	7	1,174	3.59 (3.09, 4.09)	99.0	< 0.0001
North America	3	278	4.02 (3.67, 4.36)	92.2	< 0.0001
Australia	2	134	3.78 (3.65, 3.90)	0	0.588
Recruitment time					
2010–2019	6	987	3.59 (3.15, 4.03)	98.7	< 0.0001
2020–2024	9	2,608	3.24 (2.98, 3.50)	99.0	< 0.0001
Mean age of caregivers					
< 60 years	15	3,174	3.44 (3.17, 3.71)	99.0	< 0.0001
≥ 60 years	13	1,673	3.57 (3.29, 3.84)	98.5	< 0.0001
Gender of caregivers					
Female < 70%	13	3,163	3.33 (3.08, 3.58)	99.1	< 0.0001
Female ≥ 70%	16	2,178	3.64 (3.40, 3.88)	98.0	< 0.0001
Main caregivers					
Adult child < 50%	13	2,078	3.47 (3.19, 3.74)	98.8	< 0.0001
Adult child ≥ 50%	15	3,026	3.55 (3.30, 3.81)	98.8	< 0.0001
Scales					
PACS	17	3,436	3.35 (3.17, 3.52)	98.2	< 0.0001
CSS	7	1,207	3.88 (3.57, 4.19)	97.1	< 0.0001
Other scales	5	698	3.50 (2.85, 4.15)	99.3	< 0.0001

Included studies using scales with item rating 1–5 points; CSS: Caregiving satisfaction scale; PACS: Positive aspects of caregiving scale; other scales: appraisal of caregiving scale, benefit finding scale, carers of older people in Europe index, family appraisal of caregiving questionnaire.

### 3.6 Sensitivity analysis and publication bias

After removing each included study in turn, sensitivity analyses indicated no significant changes in the pooled mean scores of PAC measured by scales using item rating 1–5 points, 0–4 points, and 1–4 points (Supplementary Figures 4–6). According to the Begg’s test and Egger’s test, no significant publication bias was observed for mean scores of PAC measured by scales using item rating 1–5 points (*p* = 0.694 and 0.479), 0–4 points (*p* = 1.000 and 0.343), and 1–4 points (*p* = 0.296 and 0.370).

### 3.7 Quality of evidence assessment results

According to the GRADE system, pooled mean scores of PAC measured using scales with item ratings of 1–5 points, 0–4 points, 1–4 points, and 0–3 points showed very low quality (Supplementary Table 3). The downgrading was attributed to high heterogeneity.

### 3.8 Associated factors of PAC

Fifteen (44.1%) studies reported factors associated with PAC (Table 4). Based on the Stress Process Model, these factors were summarized as socio-demographic factors, primary stressors, secondary stressors, and social factors.

**Table 4:** Factors associated with positive aspects of caregiving in the included studies.

Author (year)	Socio-demographic factors	Primary stressors	Secondary stressors	Social factors
Cheng et al. (29)	-	-	Self-efficacy (β = 0.186^*^)	-
Gonçalves-Pereira et al. (44)	Education (β = −0.253^*^)	-	Caregiving burden (β = −0.246^*^)	-
Hou et al. (10)	-	Physical function^a^ (*β* = −0.037^*^)	-	-
Iecovich (27)	-	Morbidity^a^ (β = 0.14^**^)	Frequency of visits (β = 0.16^**^), quality of relationships (*β* = 0.37^***^)	-
Imasio (41)	Male caregiver (OR = 0.44^*^)	Daughter-in-law caregiver (OR = 0.29^**^), caregiving obligation (OR = 0.29^*^)	-	-
Jiang et al. (11)	Financial state (β = 0.257^***^)	-	Daily caregiving time (β = 0.174^***^), good self-related health (β = 0.157^**^)	-
Liu et al. (45)	-	-	-	Familism (β = 0.347^***^), social support (β = 0.252^***^)
Liu and Sun (15)	Employment status (OR = 3.245^*^)	-	Cognitive reappraisal (OR = 1.323^***^)	Family function (OR = 1.206^**^)
López et al. (36)	Work outside (β = −0.207^**^)	Own initiative caregiver (β = 0.249^**^)	Previous affective relationship (β = 0.337^***^), venting coping (β = −0.336^***^)	-
McAuliffe et al. (32)	-	-	Caregiving burden (B = −0.48^***^)	-
Sugihara and Sugisawa (24)	-	-	-	Informal support (β = 0.07^*^)
Tomita et al. (16)	-	-	Poor mental health (B = −8.2^***^)	formal care quality (OR = 12.8^***^)
Yu et al. (7)	-	Moderate disability^a^ (β = −0.13^**^)	Poor self-related health (β = −0.18^**^)	Social support (β = 0.20^**^)
Wang et al. (47)	Education (β = 0.320^***^)	-	-	Social network type (β = 0.228*)
Zhang et al. (35)	-	Physical impairment^a^ (β = 0.091^*^)	Self-efficacy (β = 0.299^***^)	-

a Care-recipients; ^*^p < 0.05, ^**^p < 0.01, ^***^p < 0.001.

#### 3.8.1 Socio-demographic factors

Six studies reported socio-demographic factors, including gender, education level, employment status, and economic status. Male caregivers reported lower PAC than female caregivers (41). One study discovered higher PAC among caregivers with higher education level (47), while another study reported lower PAC in more educated caregivers (44). Regarding employment status, López et al. (36) found a negative association between caregivers working outside the home and their PAC, whereas Liu and Sun (15) reported a positive association between employment status and PAC. Furthermore, higher economic status was associated with greater PAC (11).

#### 3.8.2 Primary stressors

Six studies found several primary stressors. Higher levels of PAC were found in care recipients with lower physical function (10) and moderate disability (7). Conversely, two studies revealed positive associations between PAC with care recipients’ comorbidity (27) and physical impairment (35). Own initiative caregivers had a higher level of PAC (36), whereas daughter-in-law caregivers and those with a caregiving obligation had lower levels of PAC than other caregivers (41).

#### 3.8.3 Secondary stressors

Ten studies revealed eight secondary stressors. For role stressors, caregivers’ subjective burden was negatively associated with their PAC (32, 44). Greater PAC was found in caregivers who reported more frequent visits (27) and longer daily caregiving time (11). The relationship quality between caregivers and care recipients was positively related to PAC (27, 36). Caregivers with better self-rated health reported higher levels of PAC (7, 11, 16). Regarding internal stressors, caregivers’ self-efficacy (29, 35) and cognitive reappraisal (15) were positively associated with their PAC. Caregivers who used more venting coping tended to have lower PAC (36).

#### 3.8.4 Social factors

Six studies identified social factors. Higher levels of PAC were linked to greater social support (7, 45), informal support from relatives or others (24), and formal support from healthcare professionals (16). As for social network types, caregivers in a family-dominant network had higher PAC than those in family-limited or diverse social networks (47). Besides, PAC was significantly associated with familism values (45) and family function (15).

## 4 Discussion

PAC was essential for the psychological health of family caregivers and the quality of informal care for older adults. This systematic review was the first to estimate the level and associated factors of PAC among family caregivers of older adults based on 34 studies across 12 countries. This review revealed a moderate level of PAC and a variety of multifaceted factors associated with PAC. The findings can provide a better understanding of PAC and its associated factors, contributing to developing effective interventions or supportive policies for family caregivers of older adults.

In this review, more than half of the studies were published in the past five years, indicating an increasing emphasis on the assessment of PAC. The pooled estimates indicated a moderate level of PAC among family caregivers of older adults, lower than that of family caregivers of stroke survivors (48) and cancer patients (49). Family caregivers of older adults have to handle a wide range of physical tasks, emotional and behavioral symptoms, and even some medical care tasks within the home environment (24, 39). Adapting to this process is challenging. Most existing support interventions for family caregivers have emphasized reducing the negative experience of caregiving, such as caregiving burden (2). PAC could buffer the negative experience of caregiving (6). Our review highlights the necessity of support interventions aimed at enhancing PAC for family caregivers of older adults.

Subgroup analyses revealed that levels of PAC varied by geographical region, recruitment time, caregiver demographics, and assessment tools. Family caregivers of older adults in Asian countries experienced lower PAC than those in North American, European, and Australian countries. The reason may be that North American and European countries emphasize community-based services for older adults, and their highly supportive systems for family caregivers may enhance caregivers’ positive experience (6). The pooled level of PAC was lower among caregivers during 2020–2024 compared to that before 2020. The reduced level may be due to the remarkable increase in care needs and intensity of older adults caused by the COVID-19 pandemic since 2020. Regarding caregiver demographics, higher levels of PAC were observed among older, female, and adult-child caregivers. A prior review also found that older caregivers were more likely to have higher PAC than younger ones (50). A longitudinal study indicated that adult–child caregivers experienced less subjective burden and physical and mental health problems than spousal caregivers (51), which may increase their perceptions of positive benefits. Additionally, several validated scales were found to measure PAC among family caregivers. The pooled level of PAC was lower in studies that used the PACS than in those using other scales. This may be owing to differences in scale properties and cultural contexts. The complex nature of caregiving makes it difficult to measure caregivers’ positive experience (6). The influence of scales on the measurement of PAC deserves attention in future research.

Identification of factors relating to PAC is particularly important to develop targeted interventions. Based on the Stress Process Model, this review found that PAC resulted from the influence of multifaceted factors, such as socio-demographic factors, primary stressors, secondary stressors, and social factors. For socio-demographic factors, male caregivers were less likely to perceive PAC. The subgroup analysis based on gender also showed lower PAC in studies with more male caregivers. This may be explained by the differences in coping strategies across genders and caregiving contexts (41). Nevertheless, existing literature has not yet reached a consensus on the relationship between gender and PAC (34, 50). Family caregivers with better economic status could afford to satisfy basic needs and have fulfilling lives, thus perceiving a more positive experience (11). Caregivers’ education level and employment status were controversial factors of PAC, needing further investigation. In view of the above socio-demographic factors, special attention should be paid to family caregivers who are prone to have lower PAC.

Regarding primary stressors, the care recipient’s health condition may be the main objective stressor for caregivers. However, the included studies showed conflicting results on the relationship between this condition and PAC. This suggests the need for future research to explore potential moderators that could explain these conflicting findings. Besides, the obligation for the family could affect caregivers’ positive experience. Being caregivers by one’s own initiative was found to positively influence PAC. On the contrary, caring for older adults due to caregiving obligations could decrease caregivers’ rewarding experiences. These findings indicate that primary stressors warrant attention in the design of supportive interventions.

Our review found that included studies focused more on secondary stressors. Among the role stressors, subjective burden showed a negative association with PAC (32, 44). However, higher objective burden, such as more frequent visits to older adults and longer daily caregiving time, was positively associated with PAC (11, 27). These results suggest that, compared with objective burden, subjective burden may have a greater impact on caregivers’ lives and more strongly reduce their caregiving satisfaction. In addition, the better the self-rated health, the higher the levels of PAC. A possible reason is that caregivers with better health status may have more competence when facing the physical responsibility of caregiving (11). Also, a good dyadic relationship showed a positive association with PAC, similar to previous studies on family caregivers of individuals with mental illness (52). With respect to internal stressors, self-efficacy was demonstrated as an important factor of PAC, supported by previous studies on informal caregivers of cancer patients (34). As a positive psychology characteristic, high self-efficacy may increase family caregivers’ confidence in dealing with caregiving challenges and help them positively adapt to their caregiving roles (29, 35). Family caregivers who adopted negative coping strategies tended to have lower levels of PAC. Conversely, caregivers who used cognitive reappraisal tended to report higher PAC, which is in line with findings on family caregivers of dementia patients (3). Cognitive reappraisal, an emotion regulation strategy, could change the way caregivers think about distressful caregiving situations and guide them to adopt positive coping behaviors, thus increasing their sense of gain (15). Given the modifiable nature of secondary stressors, they may serve as important components of intervention programs on PAC.

Social factors are key external moderating factors that influence PAC. Among them, social support was an important factor in PAC. Previous studies also confirmed the positive association between social support and PAC among caregivers of individuals with cancer (34) and dementia (3). Caregivers in family-dominant social networks had stronger links to their family members and exhibited greater PAC (47). Better family function could offer family caregivers adequate and timely assistance, contributing to more positive feelings about their caregiving roles (15). Familism, a cultural value, emphasizes the commitment and responsibility of caring for family members (30). Caregivers holding a higher value of familism may perceive caring for older adults as a natural extension of family life and feel more benefits during the caregiving process (45). Similarly, a previous study on informal caregivers revealed the positive effect of familism on caregiving outcomes and suggested family-centered interventions to bolster positive appraisals (30). Collectively, support from healthcare professionals, families, friends, and neighbors could enable family caregivers to handle caregiving difficulties and enhance their psychological strength and adaptability (7, 16). Consequently, interventions targeting these social factors may be more effective in helping caregivers find value in their caregiving role.

### 4.1 Implications

Family caregivers are a core force in the long-term care system. Enhancing their PAC can contribute to caregiver health, care sustainability, and the resilience of long-term care systems. This review highlights the need for more attention on promoting PAC among family caregivers of older adults, especially among Asian and younger caregivers. First, assessing PAC can help healthcare professionals screen vulnerable caregivers and implement interventions to support them. Routine PAC assessment could be integrated into national or regional health surveys as a public health indicator to monitor caregiver well-being. Validated and widely used scales should be chosen to measure PAC and facilitate comparisons. Second, primary care centers should implement caregiving support interventions to enhance PAC. Psychosocial strategies could be adopted, such as cognitive behavioral therapy, mindfulness-based therapy, benefit-finding interventions, and peer support (2, 3). The use of information technology (e.g., online platforms and mobile applications) may further enhance support for family caregivers during intervention delivery (4). To maximize intervention effectiveness, multifaceted and modifiable factors, such as subjective burden, dyadic relationship, self-efficacy, coping strategies, family function, and social support, could be incorporated as components of these interventions. Embedding such services into primary care can make caregiver support more accessible and systematic. Third, policies such as paid caregiver leave, respite services, and long-term care insurance may serve as structural interventions that promote PAC by reducing burden and offering tangible recognition. Fourth, future research should further explore modifiable factors to enrich the understanding of associated factors with PAC and identify potential components for intervention programs. Longitudinal studies are also necessary to examine the progression of PAC during the caregiving process and establish causal relationships between PAC and its associated factors.

### 4.2 Limitations

This review has several limitations. First, exclusion of non-English studies may potentially overlook relevant studies in other languages and introduce language bias. Second, more than three-fifths of the included studies were from Asian countries, and this may influence the sample representativeness. Third, considerable heterogeneity was present in the pooled results. Some of the heterogeneity may be explained by differences in geographical region, recruitment time, caregiver characteristics, and assessment tools. Fourth, due to heterogeneity in assessment tools, data reporting formats, and limited statistical data across the included studies, a quantitative meta-analysis for associated factors could not be conducted, which also precluded a standard GRADE assessment for these factors. Future primary studies with larger sample sizes and more consistent reporting of relevant factors are warranted to enable such analysis and allow for assessment of the quality of evidence. Finally, the included studies employed a cross-sectional design to examine the relationships between PAC and associated factors. Future studies using a longitudinal design are therefore needed to establish causal relationships.

## 5 Conclusion

This systematic review indicated that PAC was at a moderate level among family caregivers of older adults and needed to be improved, especially for Asian and younger caregivers. Moreover, PAC can be influenced by socio-demographic factors, primary stressors, secondary stressors, and social factors. The findings call for greater attention to PAC among family caregivers of older adults. Future research should focus on exploring more multifaceted factors associated with PAC and developing targeted intervention programs based on these factors.

## Data Availability

The original contributions presented in the study are included in the article/Supplementary material, further inquiries can be directed to the corresponding author.
