# Supplementary material for: Levels of positive aspects of caregiving and associated factors among family caregivers of older adults: a systematic review and meta-analysis
Source: Front Public Health. 2026 Jun 12;14:1844907. doi: 10.3389/fpubh.2026.1844907 (PMC13303032; doi:10.3389/fpubh.2026.1844907)
Supplement: Supplementary file 1 [file Table_1.DOCX]

Supplementary Table 1 Search strategy.

| **Database** | **Search strategy** |
| --- | --- |
| PubMed | #1 ("positive aspect* of caregiving" OR "positive aspect* of care" OR "positive aspect* of caring" OR "caregiver gain*" OR "benefit finding" OR "benefit appraisal*" OR "caregiving benefits" OR "positive appraisal* of care*" OR "caregiving appraisal*" OR "caregiver appraisal*" OR "positive caregiving experience*" OR "positive experience* of caregivers" OR "positive experience* of caregiving" OR "positive feelings of care*" OR " positive perception* of caregiving " OR "caregiving gain*" OR "caregiving satisfaction") [Title/Abstract]  #2 (caregivers) [Mesh Terms] OR (caregiver* OR carer*) [Title/Abstract]  #3 #1 AND #2 AND (English [Filter]) |
| CINAHL | #1 ("positive aspect* of caregiving" OR "positive aspect* of care" OR "positive aspect* of caring" OR "caregiver gain*" OR "benefit finding" OR "benefit appraisal*" OR "caregiving benefits" OR "positive appraisal* of care*" OR "caregiving appraisal*" OR "caregiver appraisal*" OR "positive caregiving experience*" OR "positive experience* of caregivers" OR "positive experience* of caregiving" OR "positive feelings of care*" OR " positive perception* of caregiving " OR "caregiver gain*" OR "caregiving satisfaction") AB  #2 (caregiver* OR carer*) AB  #3 #1 AND #2 AND (English)LA |
| Embase | #1 ("positive aspect* of caregiving" OR "positive aspect* of care" OR "positive aspect* of caring" OR "caregiver gain*" OR "benefit finding" OR "benefit appraisal*" OR "caregiving benefits" OR "positive appraisal* of care*" OR "caregiving appraisal*" OR "caregiver appraisal*" OR "positive caregiving experience*" OR "positive experience* of caregivers" OR "positive experience* of caregiving" OR "positive feelings of care*" OR " positive perception* of caregiving " OR "caregiver gain*" OR "caregiving satisfaction") ti,ab,kw  #2 (caregiver* OR carer*) ti,ab,kw  #3 #1 AND #2 [English]/lim |
| Web of Science | #1 ("positive aspect* of caregiving" OR "positive aspect* of care" OR "positive aspect* of caring" OR "caregiver gain*" OR "benefit finding" OR "benefit appraisal*" OR "caregiving benefits" OR "positive appraisal* of care*" OR "caregiving appraisal*" OR "caregiver appraisal*" OR "positive caregiving experience*" OR "positive experience* of caregivers" OR "positive experience* of caregiving" OR "positive feelings of care*" OR " positive perception* of caregiving " OR "caregiver gain*" OR "caregiving satisfaction") ab  #2 (caregiver* OR carer*) ab  #3 #1 AND #2 AND (English) la |

Supplementary Table 2 Results of methodological quality of included studies.

| **Study** | **Item 1** | **Item 2** | **Item 3** | **Item 4** | **Item 5** | **Item 6** | **Item 7** | **Item 8** | **Item 9** | **Item 10** | **Item 11** | **Total score** |
| --- | --- | --- | --- | --- | --- | --- | --- | --- | --- | --- | --- | --- |
| Alonso et al. (2017) | Y | Y | N | UC | Y | Y | UC | Y | UC | N | UC | 5 |
| Chan et al. (2023) | Y | Y | Y | Y | Y | Y | UC | Y | UC | Y | UC | 8 |
| Chaudhry et al. (2025) | Y | Y | Y | Y | UC | Y | UC | Y | UC | Y | UC | 7 |
| Cheng et al. (2013) | Y | Y | N | Y | UC | Y | Y | Y | UC | Y | UC | 7 |
| Gonçalves-Pereira et al. (2010) | Y | Y | N | Y | Y | Y | UC | Y | UC | N | UC | 6 |
| Huo et al. (2025) | Y | Y | Y | Y | UC | Y | UC | Y | Y | N | UC | 7 |
| Iecovich (2011) | Y | N | N | UC | Y | Y | UC | Y | UC | Y | UC | 5 |
| Imasio (2015) | Y | Y | Y | Y | UC | Y | Y | Y | UC | Y | UC | 8 |
| Jiang et al. (2020) | Y | Y | Y | Y | UC | Y | UC | Y | Y | N | UC | 7 |
| Kajiwara et al. (2015) | Y | N | Y | Y | Y | Y | Y | Y | UC | Y | UC | 8 |
| Lee and Singh (2010) | Y | Y | N | UC | Y | Y | UC | Y | UC | Y | UC | 6 |
| Liu et al. (2012) | Y | Y | N | UC | Y | Y | UC | Y | UC | N | UC | 5 |
| Liu and Sun (2024) | Y | Y | Y | Y | Y | Y | UC | Y | UC | N | UC | 7 |
| Liu et al. (2022) | Y | Y | Y | Y | UC | Y | UC | Y | UC | Y | UC | 7 |
| López et al. (2005) | Y | Y | N | Y | UC | Y | UC | Y | UC | N | UC | 5 |
| López-Martínez et al. (2025) | Y | Y | Y | Y | Y | Y | UC | Y | UC | N | UC | 7 |
| Lou et al. (2024) | Y | Y | Y | Y | Y | Y | Y | Y | UC | N | UC | 8 |
| McAuliffe et al. (2020) | Y | Y | N | N | UC | Y | Y | Y | Y | N | UC | 6 |
| Narayan et al. (2001) | Y | Y | N | Y | UC | Y | UC | Y | UC | N | UC | 5 |
| Sabatini et al. (2023) | Y | Y | Y | Y | UC | Y | UC | Y | UC | N | Y | 7 |
| Schulz et al. (2024) | Y | Y | N | Y | UC | Y | UC | Y | UC | N | UC | 5 |
| Schwarz (1999) | Y | Y | N | Y | Y | Y | UC | Y | UC | N | UC | 6 |
| Schwarz and Elman (2003) | Y | Y | N | Y | Y | Y | UC | Y | UC | N | UC | 6 |
| Soskolne et al. (2007) | Y | Y | Y | Y | UC | Y | UC | Y | UC | Y | UC | 7 |
| Sugihara and Sugisawa (2023) | Y | Y | Y | Y | UC | Y | UC | Y | Y | Y | UC | 8 |
| Toljamo et al. (2012) | Y | Y | Y | Y | UC | Y | UC | Y | Y | Y | UC | 8 |
| Tomita et al. (2025) | Y | Y | Y | Y | UC | Y | UC | Y | Y | Y | UC | 8 |
| Wang et al. (2024) | Y | Y | Y | Y | UC | Y | UC | Y | N | N | UC | 6 |
| Wong et al. (2019) | Y | Y | N | Y | Y | Y | UC | Y | UC | N | UC | 6 |
| Wu et al. (2022) | Y | Y | N | Y | Y | Y | Y | Y | UC | N | UC | 7 |
| Yan et al. (2024) | Y | Y | Y | Y | UC | Y | UC | Y | UC |  | UC | 6 |
| Yu et al. (2025) | Y | Y | Y | Y | UC | Y | UC | Y | Y | Y | UC | 8 |
| Zhang et al. (2024) | Y | Y | Y | Y | UC | Y | Y | Y | Y | N | UC | 8 |
| Zhang et al. (2025) | Y | UC | Y | Y | UC | Y | UC | Y | UC | N | UC | 5 |

Y:yes; N:no; UC: unclear; Item 1: Define the source of information; Item 2: List inclusion and exclusion criteria for exposed and unexposed subjects (cases and controls) or refer to previous publications; Item 3: Indicate time period used for identifying patients; Item 4: Indicate whether or not subjects were consecutive if not population-based; Item 5: Indicate if evaluators of subjective components of study were masked to other aspects of the status of the participants; Item 6: Describe any assessments undertaken for quality assurance purposes (e.g., test/retest of primary outcome measurements); Item 7: Explain any patient exclusions from analysis; Item 8: Describe how confounding was assessed and/or controlled; Item 9: If applicable, explain how missing data were handled in the analysis; Item 10: Summarize patient response rates and completeness of data collection; Item 11: Clarify what follow-up, if any, was expected and the percentage of patients for which incomplete data or follow-up was obtained

Supplementary Table 3 Quality of evidence for pooled levels of positive aspects of caregiving.

| **Outcomes** | **No. of**  **data** | **Risk of bias** | **Inconsistency** | **Indirectness** | **Imprecision** | **Publication**  **bias** | **Evidence quality** |
| --- | --- | --- | --- | --- | --- | --- | --- |
| Pooled levels using item rating 1-5 points | 25 | 0 | -1^a^ | 0 | 0 | 0 | very low |
| Pooled levels using item rating 0-4 points | 4 | 0 | -1^a^ | 0 | 0 | 0 | very low |
| Pooled levels using item rating 1-4 points | 3 | 0 | -1^a^ | 0 | 0 | 0 | very low |
| Pooled levels using item rating 0-3 points | 2 | 0 | -1^a^ | 0 | 0 | 0 | very low |

^a^ High heterogeneity


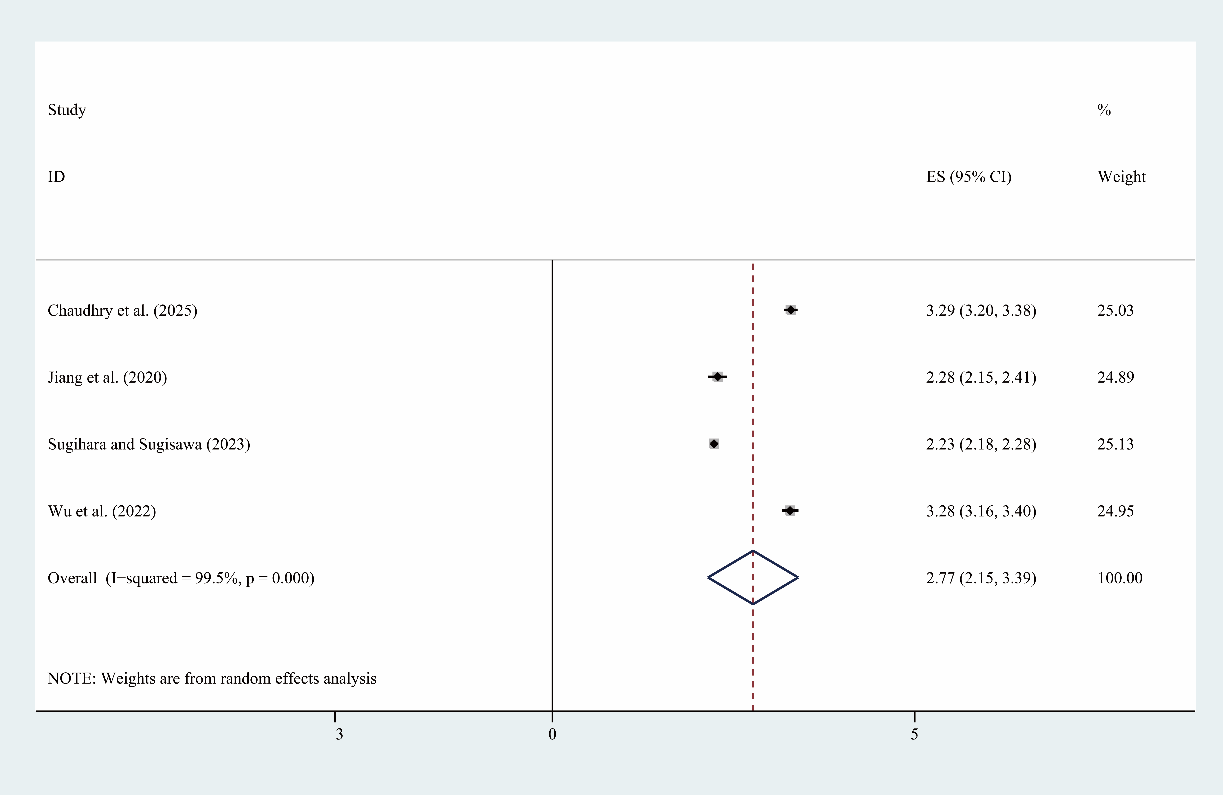


Supplementary Figure 1 Meta-analysis of mean scores of positive aspects of caregiving measured by scales using item rating 0-4 points.


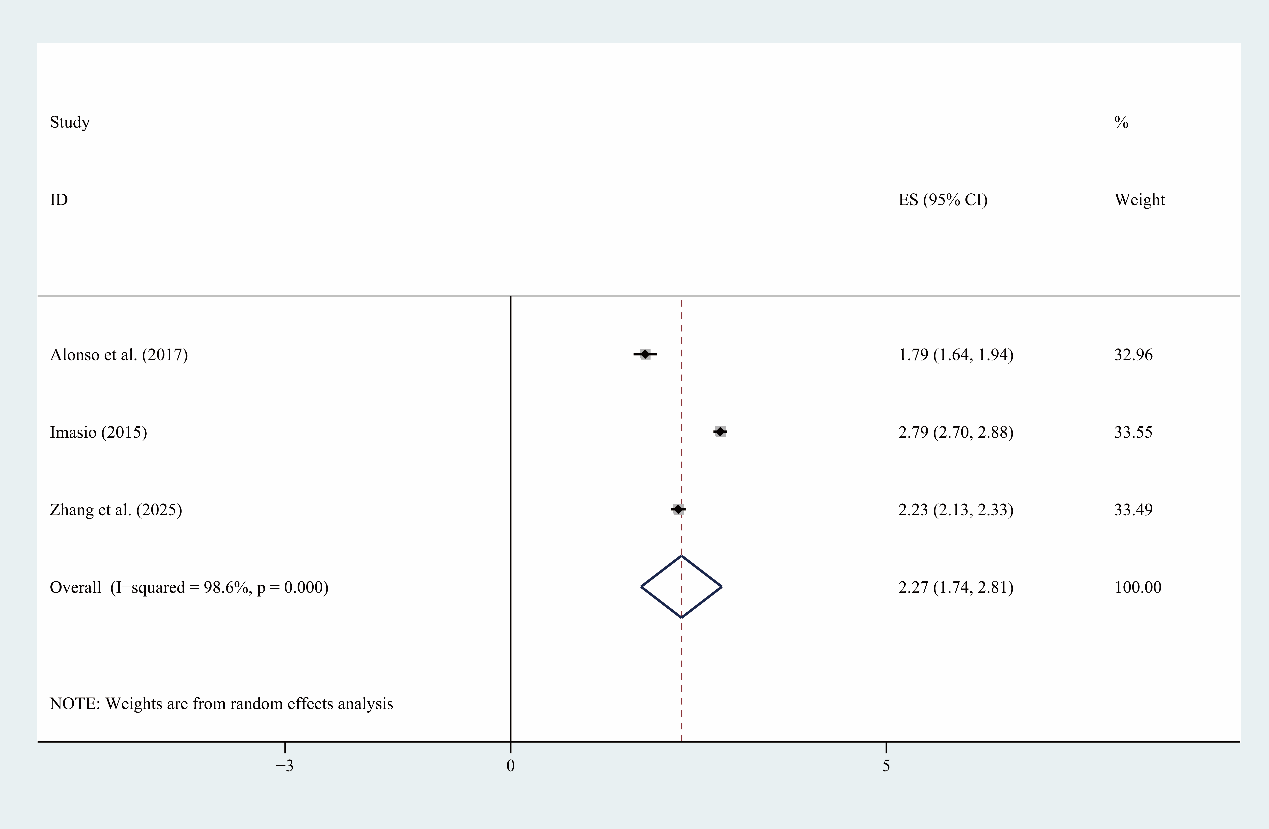


Supplementary Figure 2 Meta-analysis of mean scores of positive aspects of caregiving measured by scales using item rating 1-4 points.


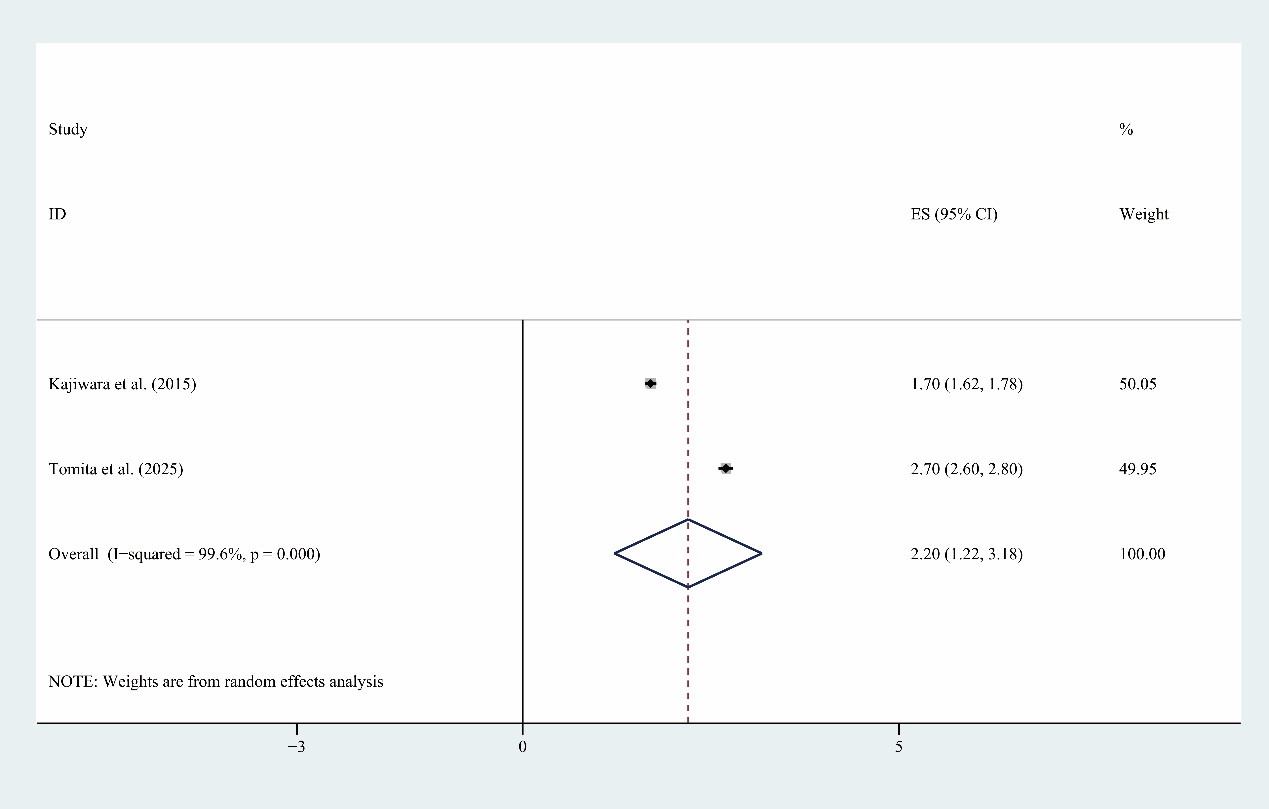


Supplementary Figure 3 Meta-analysis of mean scores of positive aspects of caregiving measured by scales using item rating 0-3 points.


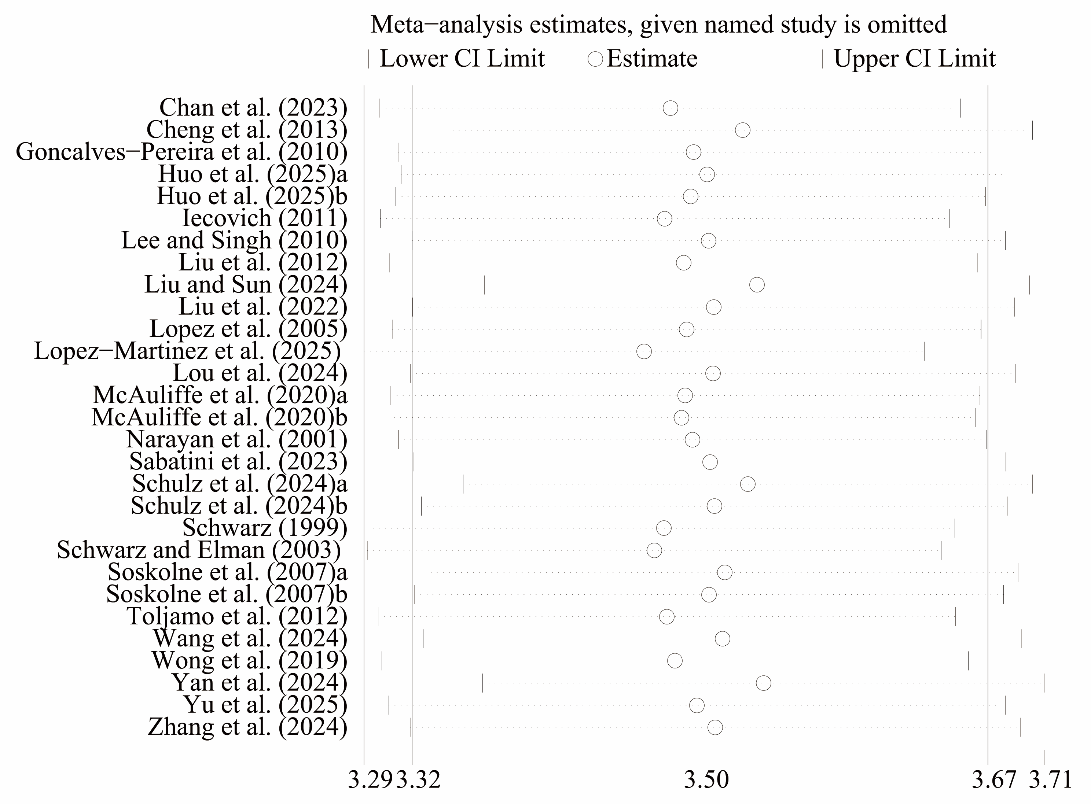


Supplementary Figure 4 Sensitivity analyses for mean scores of positive aspects of caregiving measured by scales using item rating 1-5 points.


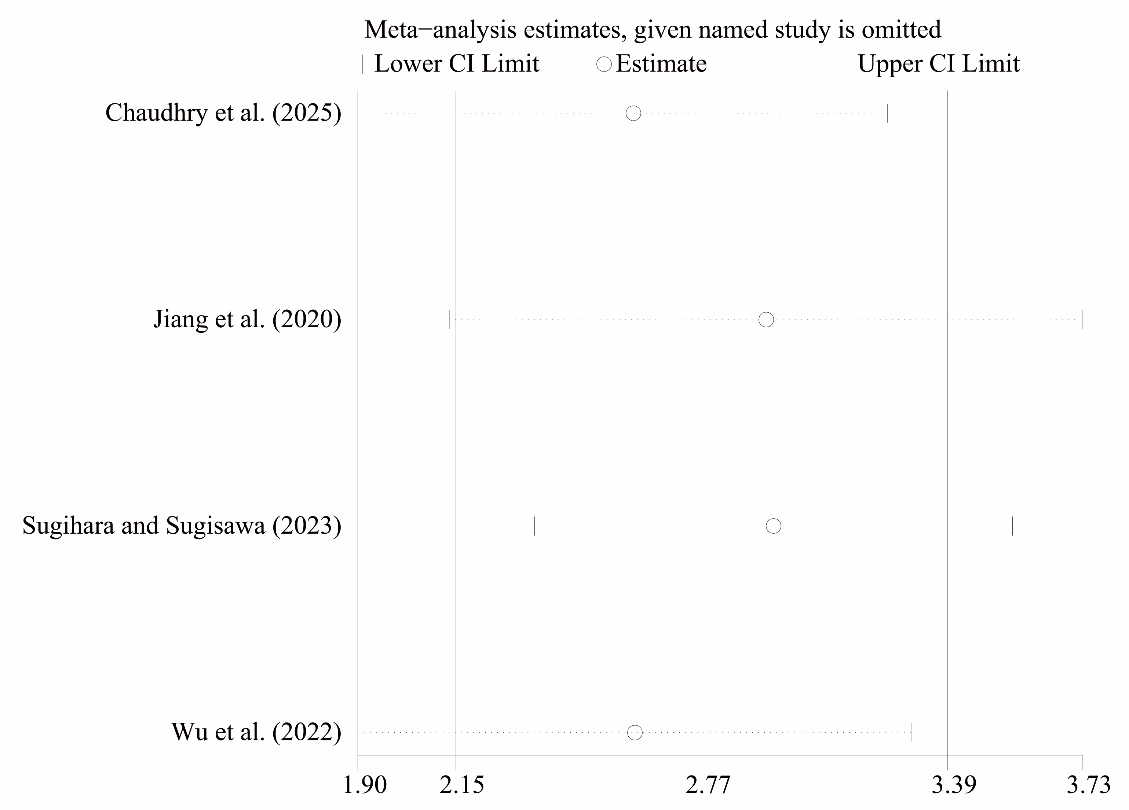


Supplementary Figure 5 Sensitivity analyses for mean scores of positive aspects of caregiving measured by scales using item rating 0-4 points.


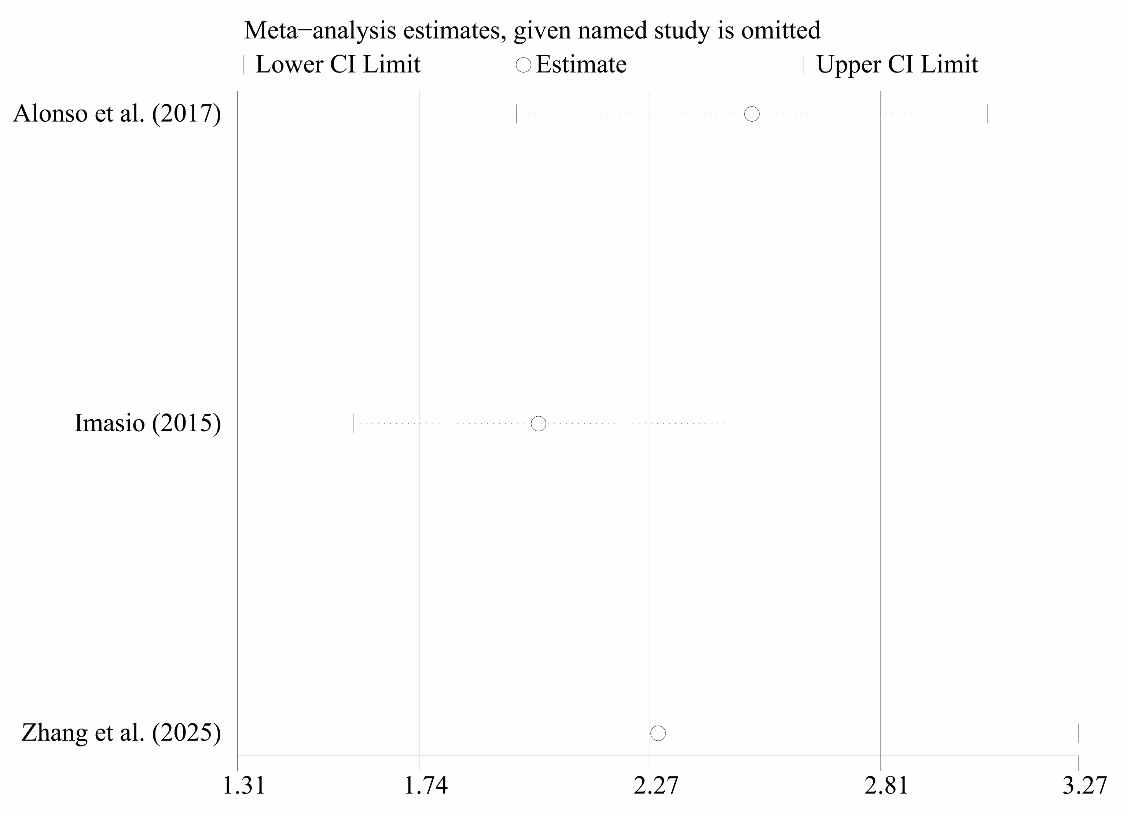


Supplementary Figure 6 Sensitivity analyses for mean scores of positive aspects of caregiving measured by scales using item rating 1-4 points.
